# Nucleotide excision repair as a targetable vulnerability in leukemia

**DOI:** 10.18632/oncotarget.22998

**Published:** 2017-12-06

**Authors:** Gregor Lohmann, Björn Schumacher, Marco Herling

**Affiliations:** Laboratory of Lymphocyte Signaling and Oncoproteome, Department of Internal Medicine I, Center for Integrated Oncology Köln-Bonn, University of Cologne, Cologne, Germany; Excellence Cluster for Cellular Stress Response and Aging-Associated Diseases and Center for Molecular Medicine Cologne, University of Cologne, Cologne, Germany

**Keywords:** CLL, nucleotide excision repair, trabectedin, DNA damage, illudins

The targeting of tumor-specific pathways in a more profound way than accom-plished by conventional cytostatics is a therapeutic challenge in many cancers, in-cluding hematologic neoplasms. In the paradigmatic entity of chronic lymphocytic leukemia (CLL), response rates after chemo-immunotherapies, e.g. based on fludar-abine-plus-antibody combinations are high, but relapses are frequent and linked with a particularly poor outcome. Despite the very impressive efficacy conferred by specific small-molecule inhibitors, such as those against BTK or BCL2, such strate-gies are also associated with incomplete clonal eradication and refractoriness or even aggressive transformation. Overall, treatment resistance becomes a concern in virtually every patient with CLL, even in the era of highly efficient chemotherapy-free agents.

Classical S-phase chemotherapeutics, such as crosslinking and double-strand break (DSB) inducing agents are of insufficient activity in CLL. Besides anti-apoptotic protection by niche-derived stimuli [1], this is largely due to a generally low replicative activity of the leukemic cell and genetically dictated deficiencies to evoke an adequate ATM/p53-mediated DNA damage response (DDR). This is particularly found in 11q23/ATM- or 17p/TP53-deleted and/or mutated high-risk CLL. In search for strategies to harness p53-independent ways of cytotoxic clearance, we have been studying non-canonical damage repair systems. We postulated that CLL cells might be more susceptible to transcription-blocking genotoxic stress. Consequently replication-independent transcription-coupled (TC) nucleotide excision repair (NER) appeared as an ideal target for CLL. Surprisingly, beyond initially promising data [2], the role of NER in CLL had not been analyzed very comprehensively.

Higher eukaryotes utilize different mechanisms for detecting and repairing potential-ly harmful or pro-tumorigenic DNA damage in actively transcribed genes and in the entire genome. The chromatin structure under low replicative activity, such as in quiescent lymphocytes, imposes constraints on the accessibility of classical (i.e. DSB) repair systems. Active transcription reduces the prevalence of somatic muta-tions accumulating in such low-access regions, particularly by utilizing NER. NER is a complex DNA repair machinery. Its two distinct recognition systems detect helix-distorting DNA lesions. Global genome (GG-) NER scans throughout the genome, while transcription-coupled (TC-) NER specifically senses lesions on the actively transcribed strand. [3,4]

**Figure 1:**
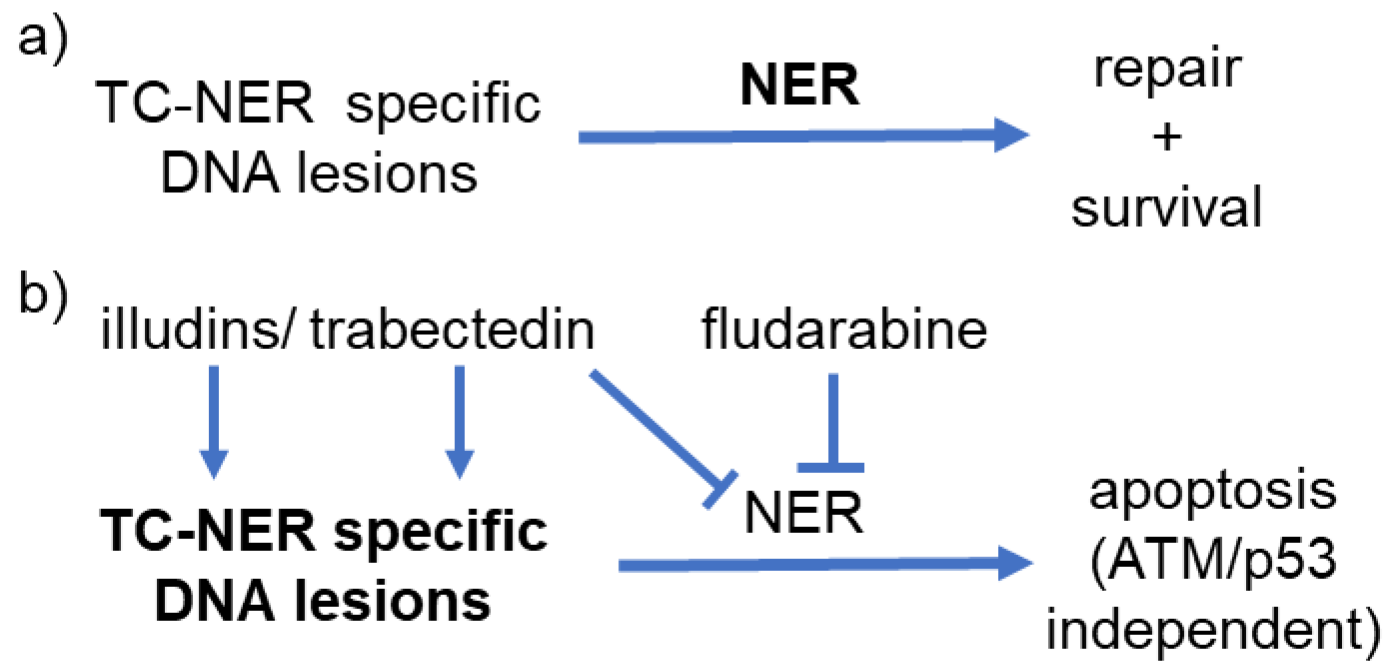
Scheme of postulated synergistic mechanisms of (TC)-NER-targeting **a** “normal scenario”: Transcription-coupled nucleotide excision repair (TC-NER) specific DNA lesions are processed by the protective NER machinery mostly re-sulting in adequate DNA repair and cell survival. **b** “scenario of ‘TC-NER-active’ compounds in synergy with fludarabine in leuke-mia”: ‘TC-NER-active‘ compounds (illudinM, ferrocen-IM or trabectedin) induce enhanced rates of TC-NER specific lesions, while nucleotide excision repair mechanisms are simultaneously blocked by trabectedin. Fludarabine poisons the final gap-filling step of the NER cascade. The ‘TC-NER-active’ agents syn-ergistically cooperate with fludarabine. This induces mainly ATM/p53 independ-ent programmed cell death and can overcome therapeutic resistance.

In Lohmann et al, Leukemia 2017 [5] we describe for the first time the association of high expression of NER genes in prospective CLL trial samples with poor response to chemo-immuntherapy. To inflict damage that requires TC-NER for removal and repair, but to potentially also induce cell death, we used illudins as they induce le-sions which are eliminated in a TC-NER specific manner (Figure 1). We performed an initial cytotoxicity screen with a large panel of illudin derivatives [6] based on genetically defined systems consisting of NER-deficient fibroblasts and of *C. elegans*, an ideal model of postmitotic tissues. It identified illudinM and ferrocen-illudinM (ferrocen-IM) as efficient and selective agents. To our panel of ‘TC-NER-active’ agents we also added the approved marine compound trabectedin, which perpetrates toxic lesions caused by TC-NER recognition of trabectedin-induced adducts (Figure 1).

The ‘TC-NER-active’ compounds (illudinM, ferrocene-IM, trabectedin) showed high activity (LD50) in freshly isolated CLL cells compared to healthy-donor PBMCs. In contrast to the chemotherapeutics fludarabine and bendamustine, these substances induced specific cell death in CLL cells irrespective of high-risk molecular profile (−11q/−17p, IGHV gene mutation status) or preceding therapy. Furthermore, the ‘TC-NER-active’ compounds conferred high cell death in samples from CLL patients who were clinically pre-exposed to fludarabine and bendamustine or even refracto-ry. The substances were also highly active in BTK inhibitor resistant cases. Experi-ments in cell systems of defined ATM deficiency and under protection by bone mar-row stroma cells corroborated the findings. Importantly, the cytotoxicity mediated by the novel ‘TC-NER-active’ agents was largely p53 independent. There were marked synergisms of the ‘TC-NER-active’ compounds with incorporated fludarabine via pre-load or simultaneous exposure, again independent of genomic aberrations like -11q or -17p. This was not observed for bendamustine.

Building on this very encouraging *in vitro* data and the well-known clinical features of trabectedin, we performed *in vivo* experiments with this agent in mouse models of CLL. In systems of syngeneic transfers of leukemic splenocytes from Eμ-TCL1 initi-ated strains, their fludarabine-refractory subclones, and their engineered TP53-deficient variants, trabectedin was active at well-tolerated dosages. It delayed the outgrowth of the engrafted leukemic clone and prolonged the survival of animals. [5]

In conclusion, because of the positive efficacy/toxicity profiles of our ‘TC-NER-active’ agents, we suggest the implementation of targeting of (TC)-NER as a salvage strategy in high-risk and refractory CLL patients or those in Richter’s transformation. As a pilot substance the well-known trabectedin appears attractive, particularly given its multi-effect profile that even goes beyond targeting NER-coupled DNA damage. [7] Its modulatory impact on the local microenvironment, particularly targeting the immunosuppressive and pro-angiogenic effects of polarized tumor-associated mac-rophages [8], is appealing with respect to ongoing efforts to convert the milieu of CLL towards a more immunogenic state.
